# Supplementary material for: HIVprotI: an integrated web based platform for prediction and design of HIV proteins inhibitors
Source: J Cheminform. 2018 Mar 9;10:12. doi: 10.1186/s13321-018-0266-y (PMC5845081; doi:10.1186/s13321-018-0266-y)
Supplement: Supplementary file 2 — Additional file 2. Source code of HIVProtI web server. [file 13321_2018_266_MOESM2_ESM.zip › HIVprotI_Source-code/hivprotI/weka-3-6-6/doc/weka/gui/sql/event/HistoryChangedEvent.html]

HistoryChangedEvent


---


|  |  |  |
| --- | --- | --- |
| **Overview** \| **Package** \| **Class** \| **Tree** \| **Deprecated** \| **Index** \| **Help** \| **Weka's home** | |  |
| **PREV CLASS**   **NEXT CLASS** | **FRAMES**    **NO FRAMES**     **All Classes** |  |
| SUMMARY: NESTED \| FIELD \| CONSTR \| METHOD | DETAIL: FIELD \| CONSTR \| METHOD |  |


---


## weka.gui.sql.event Class HistoryChangedEvent

```
java.lang.Object
  java.util.EventObject
      weka.gui.sql.event.HistoryChangedEvent
```

**All Implemented Interfaces:**: java.io.Serializable

---

``` public class HistoryChangedEvent extends java.util.EventObject ```

An event that is generated when a history is modified.

**Version:**
:   $Revision: 7059 $

**Author:**
:   FracPete (fracpete at waikato dot ac dot nz)

**See Also:**: `HistoryChangedListener`, Serialized Form

---

**Constructor Summary**

`HistoryChangedEvent(java.lang.Object source,
java.lang.String name,
javax.swing.DefaultListModel history)`
  
          constructs the event


| **Method Summary** | |
| --- | --- |
| `javax.swing.DefaultListModel` | `getHistory()`             returns the history model |
| `java.lang.String` | `getHistoryName()`             returns the name of the history |
| `java.lang.String` | `toString()`             returns the event in a string representation |

**Methods inherited from class java.util.EventObject**

`getSource`

**Methods inherited from class java.lang.Object**

`equals, getClass, hashCode, notify, notifyAll, wait, wait, wait`

**Constructor Detail**

### HistoryChangedEvent

```
public HistoryChangedEvent(java.lang.Object source,
                           java.lang.String name,
                           javax.swing.DefaultListModel history)
```

:   constructs the event

    **Parameters:**: `name` - the name of the history: `history` - the model of the history


**Method Detail**

### getHistoryName

```
public java.lang.String getHistoryName()
```

:   returns the name of the history

---


### getHistory

```
public javax.swing.DefaultListModel getHistory()
```

:   returns the history model

---


### toString

```
public java.lang.String toString()
```

:   returns the event in a string representation

    :   **Overrides:**: `toString` in class `java.util.EventObject`
    :   **Returns:**: the event in a string representation


---


|  |  |  |
| --- | --- | --- |
| **Overview** \| **Package** \| **Class** \| **Tree** \| **Deprecated** \| **Index** \| **Help** \| **Weka's home** | |  |
| **PREV CLASS**   **NEXT CLASS** | **FRAMES**    **NO FRAMES**     **All Classes** |  |
| SUMMARY: NESTED \| FIELD \| CONSTR \| METHOD | DETAIL: FIELD \| CONSTR \| METHOD |  |


---
